# Assessing Causal Relationships Between Periodontitis and Non-alcoholic Fatty Liver Disease: A Two-Sample Bidirectional Mendelian Randomisation Study

**DOI:** 10.3290/j.ohpd.b5395053

**Published:** 2024-05-28

**Authors:** Xiaofan Cheng, Jialu Chen, Siliang Liu, Shoushan Bu

**Affiliations:** a Dentist, Department of Stomatology, The First Affiliated Hospital of Nanjing Medical University, Nanjing, China. Study design, collected and analyzed the data, wrote the manuscript, reviewed and approved the final manuscript.; b Dentist, Department of Stomatology, The First Affiliated Hospital of Nanjing Medical University, Nanjing, China. Collected and analysed the data, reviewed and approved the final manuscript.; c Postgraduate Student, Department of Stomatology, The First Affiliated Hospital of Nanjing Medical University, Nanjing, China. Plotted the figures, reviewed and approved the final manuscript.; d Professor, Department of Stomatology, The First Affiliated Hospital of Nanjing Medical University, Nanjing, China. Study design, revised the manuscript, reviewed and approved the final manuscript.

**Keywords:** causality, Mendelian randomisation (MR), non-alcoholic fatty liver disease (NAFLD), periodontitis

## Abstract

**Purpose:**

To investigate the causality between periodontitis and non-alcoholic fatty liver disease (NAFLD) using a two-sample bidirectional Mendelian randomisation (MR) analysis.

**Materials and Methods:**

Genetic variations in periodontitis and NAFLD were acquired from genome-wide association studies (GWAS) using the Gene-Lifestyle Interaction in Dental Endpoints, a large-scale meta-analysis, and FinnGen consortia. Data from the first two databases were used to explore the causal relationship between periodontitis and NAFLD (“discovery stage”), and the data from FinnGen was used to validate our results (“validation stage”). We initially performed MR analysis using 5 single nucleotide polymorphisms (SNPs) in the discovery samples and 18 in the replicate samples as genetic instruments for periodontitis to investigate the causative impact of periodontitis on NAFLD. We then conducted a reverse MR analysis using 6 SNPs in the discovery samples and 4 in the replicate samples as genetic instruments for NAFLD to assess the causative impact of NAFLD on periodontitis. We further implemented heterogeneity and sensitivity analyses to assess the reliability of the MR results.

**Results:**

Periodontitis was not causally related to NAFLD (odds ratio [OR] = 1.036, 95% CI: 0.914–1.175, p = 0.578 in the discovery stage; OR = 1.070, 95% CI: 0.935–1.224, p = 0.327 in the validation stage), and NAFLD was not causally linked with periodontitis (OR = 1.059, 95% CI: 0.916–1.225, p = 0.439 in the discovery stage; OR = 0.993, 95% CI: 0.896–1.102, p = 0.901 in the validation stage). No heterogeneity was observed among the selected SNPs. Sensitivity analyses demonstrated the absence of pleiotropy and the reliability of our MR results.

**Conclusion:**

The present MR analysis showed no genetic evidence for a cause-and-effect relationship between periodontitis and NAFLD. Periodontitis may not directly influence the development of NAFLD and vice versa.

Non-alcoholic fatty liver disease (NAFLD), also known as metabolic-associated fatty liver disease (MAFLD), is currently acknowledged as the prevailing chronic liver disease globally.^14,15,32^ The prevalence of NAFLD has been documented to vary from 13.5% in Africa to 31.8% in the Middle East and continues to increase.^32^ NAFLD, characterised by abnormal or excessive fat accumulation in the liver, covers a wide range of liver conditions, from simple benign steatosis and non-alcoholic steatohepatitis (NASH) to severe liver diseases such as advanced fibrosis, cirrhosis, and hepatocellular carcinoma.^14,15,32,45^ The high incidence and severe consequences of NAFLD impose a massive burden on individuals and society, underscoring the need to reveal the underlying pathological mechanisms, investigate the risk factors for its onset and progression, and propose appropriate management strategies.^16,44^ Although the pathogenesis of NAFLD is not fully clarified, studies have shown that it is linked to inflammation and various metabolic disorders, including obesity, cardiovascular disease (CVD), type 2 diabetes mellitus (T2DM), insulin resistance, hypertension, and hyperlipidemia.^8,9^

Periodontitis, the sixth most common human disease, is a host-mediated, microbe-associated, multifactorial inflammation characterised by the progressive destruction of periodontal tissues, leading to tooth loosening or loss.^4,30^ Periodontitis negatively affects chewing function and aesthetics, and thus impairs the quality of life.^30^ Robust evidence has shown a pathophysiological relationship between periodontitis and metabolic diseases such as CVD and T2DM.^31,33^ Because NAFLD is a metabolic disease, periodontitis may also be associated with NAFLD. The association between periodontitis and NAFLD has recently attracted considerable attention. Extensive epidemiological surveys have demonstrated that NAFLD and periodontitis often occur together, and there seems to be a two-way relationship between them.^1,22,35^ On the one hand, NAFLD may aggravate the inflammation of the gingival tissue.^35^ On the other hand, periodontitis significantly increases the risk of developing NAFLD, which is proportional to the severity of periodontitis.^22^ However, the results of relevant meta-analyses conducted by Wijarnpreecha et al^41^ and Xu et al^42^ did not reveal a causative association between periodontitis and NAFLD. Investigating the causality between periodontitis and NAFLD could help us better understand the pathogenesis of these two diseases.

Mendelian randomisation (MR), a popular epidemiological analytical technique, employs genetic variants, typically single nucleotide polymorphisms (SNPs), as instrumental variables (IVs) to investigate the cause-and-effect association between exposure and outcome.^13^ MR analysis can effectively address the questions posed by confounding and reverse causality, which are inherent limitations of observational epidemiological studies, and can provide a rigorous explanation of causality between complex disorders.^10^ The MR must satisfy three hypotheses: (1) the genetic variant exhibits a strong correlation with exposure; (2) the genetic variant demonstrates independence from the confounding variable, which is correlated with both exposure and outcome; and (3) the genetic variant affects the outcome solely through exposure.^10^ This study used data from online genome-wide association studies (GWAS) to conduct a bidirectional two-sample MR analysis to investigate the causative association between periodontitis and NAFLD, hoping to offer valuable insights for clinical practice.

## Materials and Methods

### Study Design

**Fig 1:**
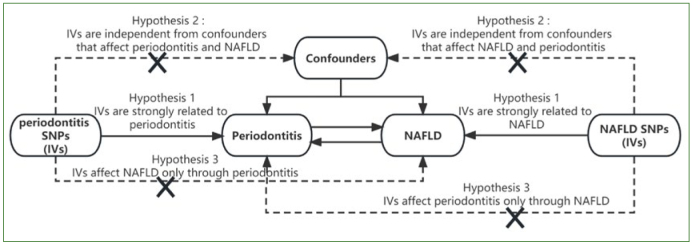
Overview of the MR design. MR, Mendelian randomisation; NAFLD, non-alcoholic fatty liver disease; SNPs, single nucleotide polymorphisms; IVs, instrumental variables.

The bidirectional relationship between periodontitis and NAFLD is summarised in Fig 1. To examine the reciprocal associations between periodontitis and NAFLD, two MR analyses were conducted using summary data obtained from GWAS. The investigation of the causal relationship between periodontitis and NAFLD can be approached from two perspectives: forward and reverse analyses. In the forward analysis, periodontitis was considered the exposure variable, while NAFLD was regarded as the outcome variable. Conversely, in the reverse analysis, NAFLD was treated as the exposure variable, and periodontitis was considered the outcome variable. Since our research relied solely on publicly accessible summary data, ethical assessment was unnecessary.

### Data Sources

The genetic variants associated with periodontitis were obtained from the Gene-Lifestyle Interaction in the Dental Endpoints Consortium (https://data.bris.ac.uk/data/dataset/2j2rqgzedxlq02oqbb4vmycnc2).^34^ The dataset consisted of 34,611 individuals of European ethnicity, excluding Hispanic/Latino individuals, comprising 12,289 periodontitis cases and 22,326 controls. The genetic variation data for NAFLD were obtained from a large-scale meta-analysis of GWAS, including 8434 cases and 770,180 controls of European ancestry.^19^ Data from these two sources were used to explore the causal relationship between periodontitis and NAFLD; this is termed “discovery stage” in the following. Other GWAS data for periodontitis and NAFLD were selected from the R9 version of FinnGen (https://r9.finngen.fi) as replicate samples to validate our results;^24^ this is hereafter termed “validation stage.” The data on periodontitis involved 4434 chronic periodontitis cases and 259,234 controls, while the data on NAFLD involved 2275 cases and 375,002 controls. All included participants were of European descent.

To establish the validity of the first MR hypothesis, SNPs were selected that exhibited a statistically significant association with periodontitis and NAFLD, with p < 5x10^-8^. Given that few SNPs for periodontitis were selected in both discovery and replicate samples, the statistical significance threshold was expanded to 5x10^-6^ to select suitable IVs for periodontitis.^38^ To ensure the independence of the SNPs, linkage disequilibrium (LD) clumping was conducted using a threshold of r^2^ < 0.001 or a clump distance greater than 10,000 kb. Each selected SNP had to possess an F-statistic (bata2/se2) greater than 10 to demonstrate a substantial correlation with the trait in the analysis.^17^ Furthermore, to validate the second hypothesis, we investigated whether SNPs exhibited independence from confounding factors at significance thresholds of p < 1x10^-5^. This analysis was performed using the Pheoscanner V2 database, accessible at http://www.phenoscanner.medschl.cam.ac.uk. The confounding factors of periodontitis and NAFLD that have been discovered are smoking and T2DM.^33,36,39,46^ Therefore, SNPs exhibiting pleiotropy and those linked to smoking or T2DM were excluded. Finally, SNPs with non-concordant alleles and intermediate allele frequencies were eliminated by harmonising the exposure and outcome datasets. As a result, five SNPs related to periodontitis and six related to NAFLD were identified in the discovery samples, whereas eighteen SNPs related to periodontitis and four related to NAFLD were identified in the replicate samples (Tables 1-4).

**Table 1:** Details of IVs after harmonising the exposure (periodontitis) and outcome (NAFLD) data in the discovery stage

SNP	EA	OA	Periodontitis				NAFLD		
			beta	se	p-value	F	beta	se	p-value
rs13005050	T	C	-0.1432	0.031	3.85E-06	21.3384	-0.0358	0.0277	0.1969
rs151226594	T	G	-0.3671	0.0768	1.75E-06	22.8479	0.0059	0.0644	0.9275
rs2976950	A	G	0.0963	0.0195	7.87E-07	24.3884	0.0094	0.0176	0.5944
rs6816769	T	C	-0.1348	0.0294	4.54E-06	21.0225	-0.0093	0.0262	0.7236
rs73155039	A	G	0.8316	0.1757	2.21E-06	22.4019	-0.0146	0.0739	0.8437

After selecting SNPs by p-value and clumping the data, eight SNPs remained. When harmonising the exposure and outcome datasets, three SNPs (rs78422482, rs4956201 and rs138868497) with intermediate allele frequencies were eliminated. There were five SNPs left. EA: effect allele; OA: other allele.

**Table 2:** Details of IVs after harmonising the exposure (periodontitis) and outcome (NAFLD) data in the validation stage

SNP	EA	OA	Periodontitis				NAFLD		
			beta	se	p-value	F	beta	se	p-value
rs10268587	G	A	-0.2947	0.0621	2.10E-06	22.5023	-0.0912	0.0797	0.2527
rs113059383	G	A	-0.8399	0.1795	2.89E-06	21.8915	-0.2175	0.1923	0.2581
rs1148464	T	C	-0.1430	0.0307	3.25E-06	21.6630	-0.0104	0.0441	0.8139
rs115120340	T	C	0.1849	0.0361	3.01E-07	26.2444	0.0515	0.0521	0.3227
rs11605185	C	T	-0.2312	0.0475	1.12E-06	23.7035	0.0477	0.0623	0.4445
rs1241497	A	G	-0.1275	0.0275	3.63E-06	21.4526	0.0111	0.0391	0.7764
rs130985	T	C	-0.3131	0.0684	4.77E-06	20.9268	0.0774	0.0872	0.3747
rs139232605	G	A	0.2213	0.0417	1.13E-07	28.1357	0.0244	0.0618	0.6926
rs141098993	A	G	0.2686	0.0568	2.25E-06	22.3692	0.0379	0.0855	0.6579
rs146734691	A	G	0.7334	0.1604	4.83E-06	20.9051	-0.3175	0.2842	0.2640
rs2847728	T	G	-0.1259	0.0271	3.33E-06	21.6188	-0.0306	0.0368	0.4055
rs35813112	G	A	0.2360	0.0494	1.79E-06	22.8119	0.1636	0.0738	0.0266
rs55875437	T	C	0.1256	0.0261	1.50E-06	23.1541	-0.0191	0.0371	0.6071
rs56265851	T	A	0.2360	0.0492	1.63E-06	22.9886	0.0003	0.0726	0.9968
rs6845106	T	C	-0.1017	0.0218	3.02E-06	21.8029	-0.0262	0.0304	0.3897
rs72682016	A	T	-0.2171	0.0464	2.89E-06	21.8882	0.0759	0.0606	0.2107
rs7629105	A	G	-0.1892	0.0407	3.26E-06	21.6547	-0.0716	0.0597	0.2307
rs9645299	C	T	0.1104	0.0242	4.92E-06	20.8668	-0.0252	0.0330	0.4460

After selecting SNPs by p-value, clumping the data and F significance value, nineteen SNPs remained. When harmonising the SNP-periodontitis and SNP-NAFLD, removing rs2220232 for being palindromic with intermediate allele frequency, eighteen SNPs remained. EA: effect allele; OA: other allele.

**Table 3:** Details of IVs after harmonising the exposure (NAFLD) and outcome (periodontitis) data in the discovery stage

SNP	EA	OA	NAFLD				Periodontitis		
			beta	se	p-value	F	beta	se	p-value
rs1397844	T	C	-0.1011	0.0212	1.79E-06	22.8063	0.0434	0.023	0.05941
rs2933904	A	G	0.0805	0.0169	1.95E-06	22.6463	-0.0084	0.0231	0.7178
rs3747207	A	G	0.2886	0.0198	5.07E-48	211.9823	0.0196	0.0214	0.3589
rs710462	T	G	-0.0759	0.0164	4.07E-06	21.2316	-0.0225	0.0182	0.2164
rs77888509	A	G	-0.1146	0.0244	2.62E-06	22.0794	-0.0498	0.0447	0.2648
rs8108364	T	C	-0.1293	0.0245	1.33E-07	27.8169	-0.0231	0.0268	0.3885

After selecting SNPs by p-value, clumping the data and F significance value, there were 12 SNPs left. Four SNPs (rs780093, rs9922619, re73001065 and rs429358) related to T2DM were removed. When harmonizing the exposure and outcome datasets, 2 SNPs (rs28601761, rs7144175) with intermediate allele frequency were eliminated. There were 6 SNPs left. EA: effect allele; OA: other allele.

**Table 4:** Details of IVs after harmonising the exposure (NAFLD) and outcome (periodontitis) data in the validation stage

SNP	EA	OA	NAFLD				Periodontitis		
			beta	se	p-value	F	beta	se	p-value
rs2745359	C	T	0.3012	0.0497	1.38E-09	36.6948	0.0106	0.0392	0.7859
rs28703824	A	G	0.2343	0.0420	2.40E-08	31.1397	0.0618	0.0323	0.0560
rs738409	G	C	0.4673	0.0320	2.17E-48	213.6732	-0.0255	0.0257	0.3221
rs8100204	A	G	0.3297	0.0378	2.65E-18	76.1318	-0.0044	0.0297	0.8832

After selecting SNPs by p-value and clumping the data, five SNPs remained. When harmonising the exposure and outcome datasets, removing rs2954029 with intermediate allele frequency, there were four SNPs left. EA: effect allele; OA: other allele.

### Statistical Analyses

Five commonly utilised data regression methods were employed for the MR analysis: MR-Egger, weighted median, inverse variance weighted (IVW), simple mode, and weighted mode methods. Among these methods, the IVW method served as the principal method. This method yields the most accurate result when all IVs are effective.^7^ The MR-Egger technique provides a consistent estimate of the causal effect using weighted linear regression even when all chosen SNPs are invalid IVs.^5^ However, this approach has a limited level of accuracy and is affected by outlying genetic variations. The use of weighted median regression can generate a reliable causal estimate with no less than 50% of the IVs being effective.^6^ The weighted median method has been confirmed to surpass the MR-Egger approach owing to its smaller type I error and stronger causal estimate ability. In terms of the simple mode, it offers robustness for pleiotropy despite its lower causal precision compared with the IVW method.^28^ The weighted mode method divides SNPs into clusters based on the similarity of causal effects, and performs the causal effect estimation of the cluster with the largest number of SNPs.^21^ The five methods are readily available in the TwoSampleMR version 0.5.7 package.

To determine the heterogeneity between the SNPs, Cochran’s Q test was conducted using the MR-Egger and IVW methods. Sensitivity analyses were carried out to assess the presence of pleiotropy. The Mendelian randomisation pleiotropy residual sum and outlier (MR-PRESSO) test was applied to discover potential outlier IVs.^37^ The MR-Egger regression was performed to assess the presence of horizontal pleiotropy.^5^ Finally, a leave-one-out test was applied to test the stability and reliability of the MR findings.

**Fig 2:**
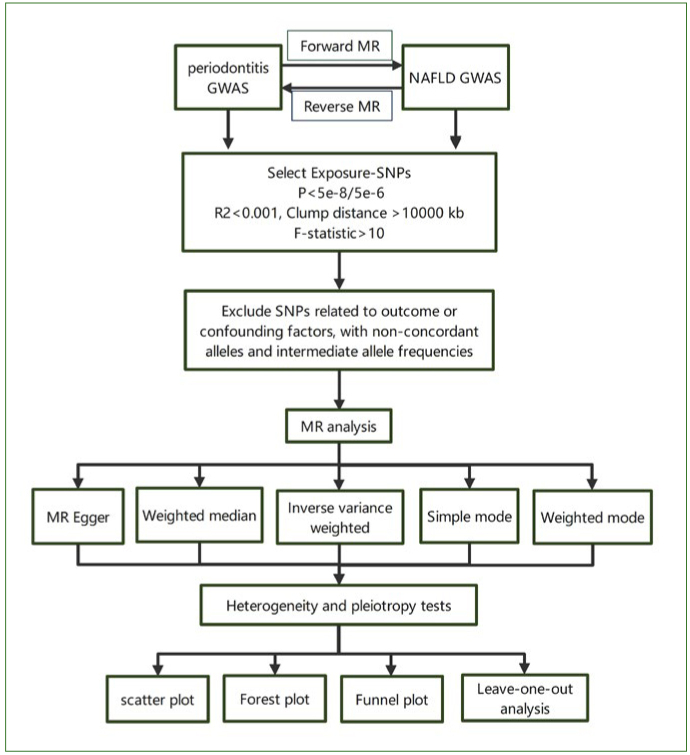
The workflow for screening of IVs and MR analysis process. GWAS, genome-wide association studies; MR, Mendelian randomisation; NAFLD, non-alcoholic fatty liver disease; SNPs, single nucleotide polymorphisms; IVs, instrumental variables.

Rstudio software, specifically R version 4.2.2, was utilised for all the analyses. The installation packages mostly consisted of TwoSampleMR version 0.5.7. Statistical significance was set at p < 0.05. A concise flowchart of this MR study is shown in Fig 2.

## Results

### The Causative Impact of Periodontitis on NAFLD in the Discovery Stage

In the analysis of the causative impact of periodontitis on NAFLD, since no SNPs related to periodontitis with p < 5x10^-8^ were identified, the statistical significance level was broadened to 5x10^-6^, and 18 SNPs statistically significantly related to periodontitis were screened out. After LD clumping, eight SNPs were retained for the analysis. The eight SNPs were not linked to smoking or T2DM, indicating independence from confounding factors. Three SNPs with intermediate allele frequencies were eliminated when the exposure and outcome datasets were harmonised. Consequently, five exposure SNPs were identified in the MR analysis (Table 1).

**Fig 3:**
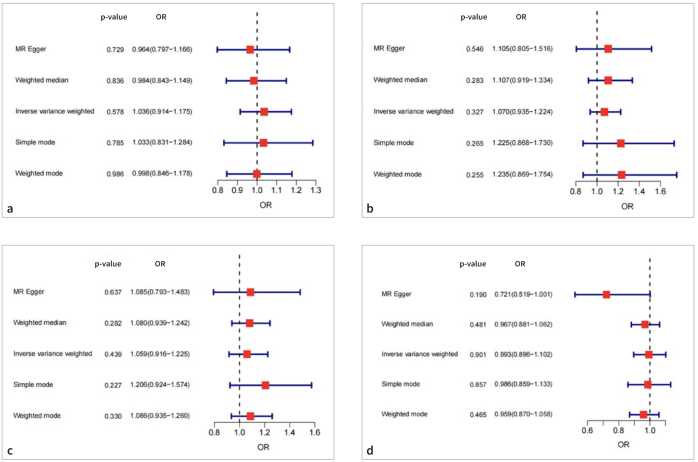
Forest plot regarding the bidirectional causal association between periodontitis and NAFLD. (a) The causal effect of periodontitis on NAFLD in the discovery stage. (b) The causal effect of periodontitis on NAFLD in the validation stage. (c) The causal effect of NAFLD on periodontitis in the discovery stage. (d) The causal effect of NAFLD on periodontitis in the validation stage. NAFLD, non-alcoholic fatty liver disease; OR, odds ratio.

**Fig 4:**
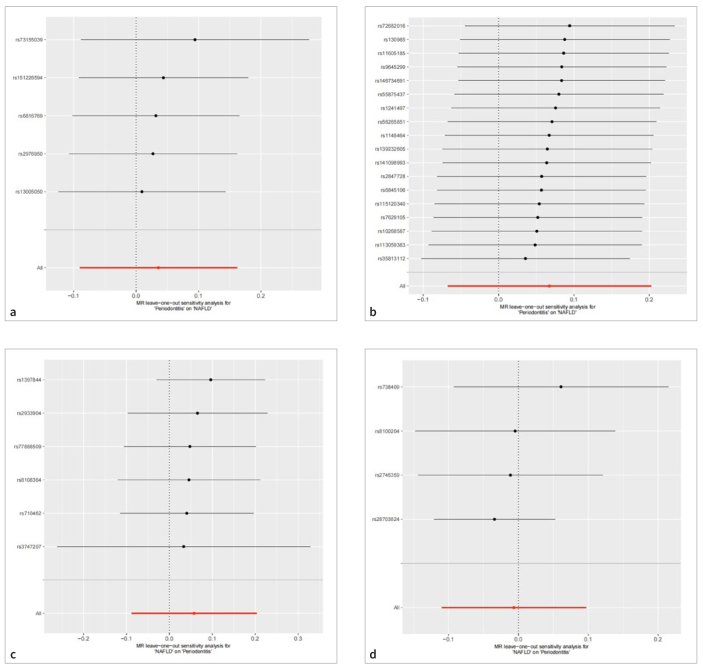
Leave-one-out analysis to examine whether the causal effect was driven by a single SNP. (a) The causative impact of periodontitis on NAFLD in the discovery stage. (b) The causative impact of periodontitis on NAFLD in the validation stage. (c) The causative impact of NAFLD on periodontitis in the discovery stage. (d) The causative impact of NAFLD on periodontitis in the validation stage. MR, Mendelian randomisation; NAFLD, non-alcoholic fatty liver disease; SNP, single nucleotide polymorphism.

As shown in Table 5 and Fig 3a, the results of the IVW (odds ratio [OR] = 1.036, 95% CI: 0.914–1.175, p = 0.578), MR-Egger (OR = 0.964, 95% CI: 0.797–1.166, p = 0.729), weighted median (OR = 0.984, 95% CI: 0.843–1.149, p = 0.836), simple mode (OR = 1.033, 95% CI: 0.831–1.284, p = 0.785), and weighted mode (OR = 0.998, 95% CI: 0.846–1.178, p = 0.986) methods were consistent, suggesting that having periodontitis was not causally related to NAFLD. Subsequently, heterogeneity and sensitivity analyses were conducted on the obtained results. The p-values of the Q-statistics of the IVW and MR-Egger methods were 0.770 and 0.846, respectively, both of which were substantially higher than 0.05, indicating no heterogeneity between the selected IVs. The MR-PRESSO global test demonstrated that no outliers required elimination (p ≥ 0.05). The intercept term of the MR-Egger regression model was 0.019 (p ≥ 0.05), indicating the absence of horizontal pleiotropy (Table 6). The leave-one-out approach did not identify any outliers that had a significant impact on the results of the MR analysis (p > 0.05) (Fig 4a).

**Table 5:** Bidirectional causal association between periodontitis and NAFLD in the discovery stage

Exposure	Outcome	MR methods	Number of SNPs	p-value	OR (95% CI)
Periodontitis	NAFLD	MR Egger	5	0.729	0.964 (0.797–1.166)
		Weighted median	5	0.836	0.984 (0.843–1.149)
		Inverse variance weighted	5	0.578	1.036 (0.914–1.175)
		Simple mode	5	0.785	1.033 (0.831–1.284)
		Weighted mode	5	0.986	0.998 (0.846–1.178)
NAFLD	Periodontitis	MR Egger	6	0.637	1.085 (0.793–1.483)
		Weighted median	6	0.282	1.080 (0.939–1.242)
		Inverse variance weighted	6	0.439	1.059 (0.916–1.225)
		Simple mode	6	0.227	1.206 (0.924–1.574)
		Weighted mode	6	0.330	1.086 (0.935–1.260)

**Table 6:** Heterogeneity and pleiotropy tests in the discovery stage

Exposure	Outcome	Heterogeneity (IVW)		Heterogeneity (MR-Egg)		Horizontal pleiotropy (MR-Egg)			MR-PRESSO (global test)
		Q	p-value	Q	p-value	Intercept	se	p-value	p-value
Periodontitis	NAFLD	1.812	0.770	0.816	0.846	0.019	0.019	0.392	0.763
NAFLD	Periodontitis	7.185	0.207	7.130	0.129	-0.004	0.025	0.870	0.407

### The Causative Impact of Periodontitis on NAFLD in the Validation Stage

In the validation phase, 89 SNPs with p-values lower than 5x10^-6^ were identified. Nineteen SNPs remained after LD clumping, and their F-statistics were higher than 10, indicating that the enrolled SNPs were strongly associated with periodontitis. When the SNP-periodontitis and SNP-NAFLD were harmonised, and rs2220232 was removed for being palindromic with an intermediate allele frequency, 18 periodontitis-related SNPs were chosen for the MR analysis (Table 2).

The results of the five MR analysis methods consistently demonstrated that periodontitis had no causative impact on NAFLD risk in the replicate samples (p > 0.05), which was consistent with the findings from the discovery samples (Table 7, Fig 3b). No heterogeneity was observed in the Cochran’s Q test of the IVW and MR-Egger methods (p > 0.05). Furthermore, the MR-PRESSO global test showed that no outliers existed (p > 0.05). According to the pleiotropy test, the intercept term of the MR-Egger was -0.0062 (p ≥ 0.05), indicating no horizontal pleiotropy (Table 8). The robustness of the obtained results was further proven using the leave-one-out approach, as no outliers were found (Fig 4b).

**Table 7:** Bidirectional causal association between periodontitis and NAFLD in the validation stage

Exposure	Outcome	MR methods	Number of SNPs	p-value	OR (95% CI)
Periodontitis	NAFLD	MR Egger	18	0.546	1.105 (0.805–1.516)
		Weighted median	18	0.283	1.107 (0.919–1.334)
		Inverse variance weighted	18	0.327	1.070 (0.935–1.224)
		Simple mode	18	0.265	1.225 (0.868–1.730)
		Weighted mode	18	0.255	1.235 (0.869–1.754)
NAFLD	Periodontitis	MR Egger	4	0.190	0.721 (0.519–1.001)
		Weighted median	4	0.481	0.967 (0.881–1.062)
		Inverse variance weighted	4	0.901	0.993 (0.896–1.102)
		Simple mode	4	0.857	0.986 (0.859–1.133)
		Weighted mode	4	0.465	0.959 (0.870–1.058)

**Table 8:** Heterogeneity and pleiotropy tests in the validation stage

Exposure	Outcome	Heterogeneity (IVW)		Heterogeneity (MR-Egg)		Horizontal pleiotropy (MR-Egg)			MR-PRESSO (global test)
		Q	p-value	Q	p-value	Intercept	se	p-value	p-value
Periodontitis	NAFLD	15.907	0.530	15.859	0.463	-0.006	0.028	0.829	0.533
NAFLD	Periodontitis	4.704	0.195	0.791	0.673	0.121	0.061	0.187	0.321

### The Causative Impact of NAFLD on Periodontitis in the Discovery Stage

In the reverse MR analysis, 407 SNPs related to NAFLD were extracted with p < 5x10^-8^. After LD clumping and the F-test (F>10), 12 SNPs remained for the analysis. Four SNPs related to T2DM and two palindromic SNPs with intermediate allele frequencies were excluded. The remaining six SNPs were used for further analyses (Table 3).

The primary IVW results (OR = 1.059, 95% CI: 0.916–1.225, p = 0.439) showed that NAFLD was not directly linked with periodontitis. Consistent conclusions were obtained using the MR-Egger, weighted median, simple mode, and weighted mode methods (Table 5, Fig 3c). Regarding heterogeneity between the selected SNPs, the p-values of the Q statistics of the IVW and MR-Egger methods were 0.207 and 0.129, respectively, indicating that no heterogeneity existed. The MR-PRESSO global test demonstrated that no outliers needed to be eliminated (p ≥ 0.05). The MR-Egger regression model had an intercept term of -0.004, with a p-value of 0.870, suggesting that horizontal pleiotropy was absent (Table 7). The leave-one-out analysis proved the stability and reliability of the analytical outcome as the cause estimate could not be changed by any single SNP (Fig 4c).

### The Causative Impact of NAFLD on Periodontitis in the Validation Stage

In the replicated reserve MR analysis, 371 SNPs related to NAFLD were extracted, with p < 5x10^-8^. Five SNPs remained after LD clumping, and their F-statistics were higher than 10. No SNP was associated with the confounding factors. One palindromic SNP (rs2954029) with an intermediate allele frequency was removed after harmonising the NAFLD and periodontitis datasets, and four NAFLD-related SNPs were ultimately screened to investigate the causal effect of NAFLD on periodontitis (Table 4).

**Fig S1:**
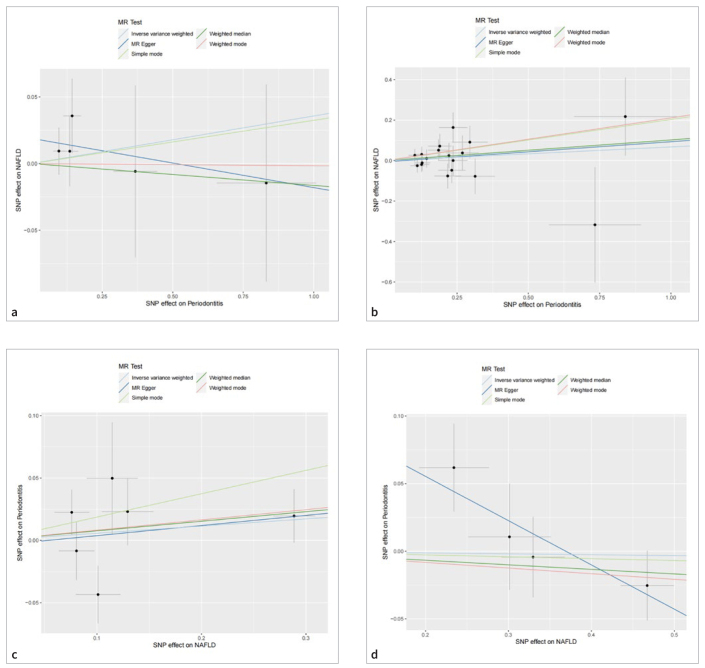
Scatter plot of the SNPs associated with periodontitis and NAFLD. a) MR estimates for periodontitis on NAFLD in the discovery stage. b) MR estimates for periodontitis on NAFLD in the validation stage. c) MR estimates for NAFLD on periodontitis in the discovery stage. d) MR estimates for NAFLD on periodontitis in the validation stage.

**Fig S2:**
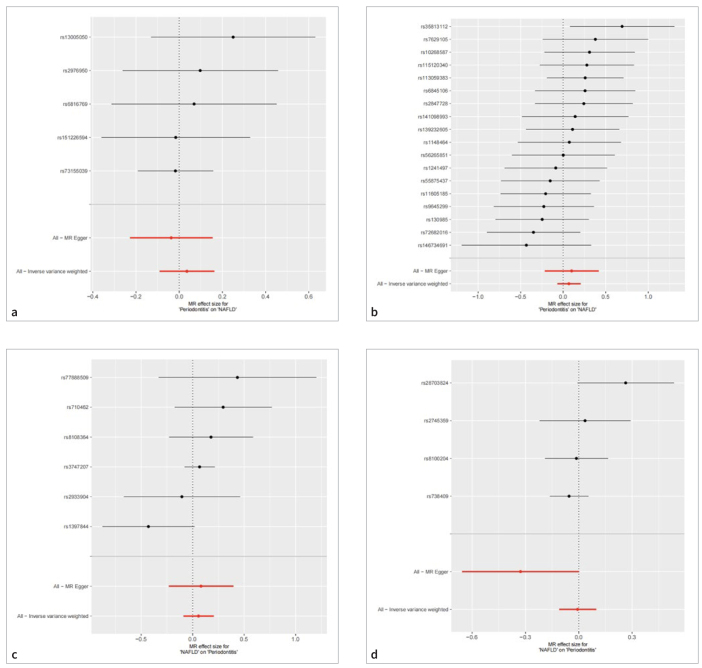
Forest plot of the SNPs associated with periodontitis and NAFLD. a) MR estimates for periodontitis on NAFLD in discovery stage. b) MR estimates for periodontitis on NAFLD in validation stage. c) MR estimates for NAFLD on periodontitis in discovery stage. d) MR estimates for NAFLD on periodontitis in validation stage.

**Fig S3:**
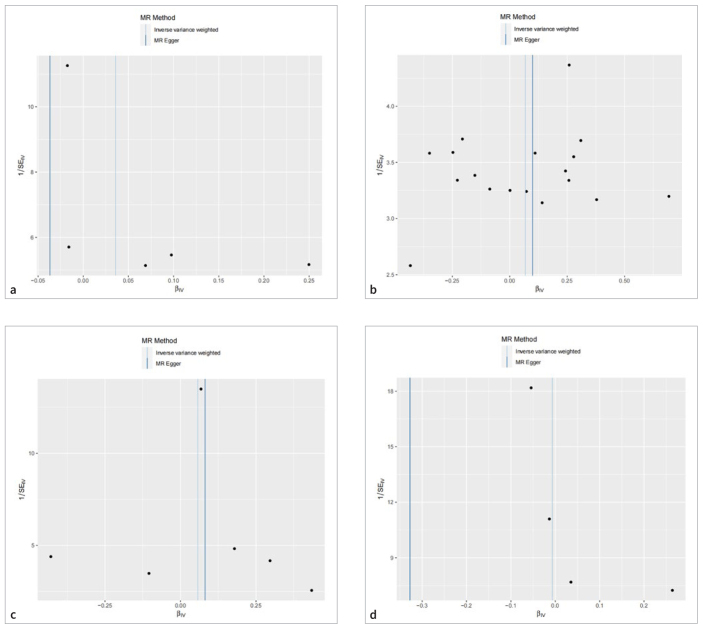
Funnel plot of the SNPs associated with periodontitis and NAFLD. a) MR estimates for periodontitis on NAFLD in the discovery stage. b) MR estimates for periodontitis on NAFLD in the validation stage. c) MR estimates for NAFLD on periodontitis in the discovery stage. d) MR estimates for NAFLD on periodontitis in the validation stage.

No causative impact of NAFLD on the risk of periodontitis was found using any of the MR analysis methods (p > 0.05) (Table 6, Fig 3d). The p-values of the Q statistics of the IVW and MR-Egger methods were 0.195 and 0.673 (i.e., > 0.05), respectively, indicating that no heterogeneity existed between the selected SNPs. The p-value of the MR-PRESSO global test was 0.321, implying that no outliers were detected. The intercept term of the MR-Egger regression model was 0.121 (p ≥ 0.05), demonstrating the absence of horizontal pleiotropy (Table 8). The leave-one-out analysis identified no outliers, suggesting that the four selected SNPs could not generate misleading results (Fig 4d). The scatter plot, forest plot, and funnel plot of the SNPs associated with periodontitis and NAFLD are depicted in the supplemental figures S1 to S3, respectively.

## Discussion

The present study is the first to examine the bidirectional causality between periodontitis and NAFLD by performing a two-sample MR analysis using two separate sets of GWAS data. No causal relationship between periodontitis and NAFLD was observed in the MR analysis.

The findings of this study did not establish a causative association between periodontitis and NAFLD, which is consistent with the results of relevant meta-analyses conducted by Wijarnpreecha et al^41^ and Xu et al,^42^ but inconsistent with the results of a number of epidemiological studies,^1,2,22,35,40^ as previously reported observational epidemiological studies were prone to confounding factors, reverse causation and other biases. Several population-based epidemiologic surveys and descriptive studies have demonstrated a correlation between the severity of periodontitis and the presence of NAFLD.^1,2,22,35,40^ In a 12-year cohort study conducted in Finland, 6165 Finnish adults were examined to investigate the association between periodontitis and incident severe liver disease. The authors found that participants with severe periodontitis exhibited a significantly elevated risk of developing NAFLD compared to those with moderate, mild, or no periodontitis.^22^ Weintraub et al^40^ investigated the correlation between NAFLD and periodontitis by analysing data from the National Health and Nutrition Examination Survey (NHANES) conducted between 1988 and 1994. A strong association was observed between the development of NAFLD and moderate and severe periodontitis when NAFLD was diagnosed using the American Fatty Liver Index and Fibrosis Score with odds ratios of 2.21 and 3.10, respectively. Alazawi et al^2^ also explored the association between NAFLD and periodontitis using data from the NHANES. They discovered that the prevalence of periodontitis in patients with NASH with significant hepatic fibrosis was 33%, which was higher than the prevalence of periodontitis in patients with NASH with no or mild hepatic fibrosis (24%). The prevalence of periodontitis was lowest in patients with NAFLD (3%), suggesting that the degree of hepatic fibrosis in patients with NAFLD is associated with the prevalence of periodontitis.^2^ Surlin et al^35^ used optical coherence tomography to assess the degree of inflammatory changes in gingival tissues and showed that patients with periodontitis and NAFLD had a lower pixel density and more inflammatory changes than those with periodontitis without NAFLD and healthy individuals, indicating that NAFLD may aggravate inflammation in periodontal disease.

There are several potential explanations for the statistically significant associations between periodontitis and NAFLD found in observational studies:

1. Periodontal pathogens may be independent common pathological causes of periodontitis and NAFLD. It has been reported that *Porphyromonas gingivalis* infection may aggravate the pathological progression of NASH in mice, causing the transition from simple steatohepatitis to steatohepatitis with fibrosis.^18^ A clinical study demonstrated a remarkably higher detection rate of *P. gingivalis* in NAFLD patients than in non-NAFLD controls. A study showed that 3 months of non-surgical periodontal treatment in patients with NAFLD improved their liver function parameters, such as serum as AST and ALT levels.^43^
2. Inflammatory mediators may be involved in the pathological processes of periodontitis and NAFLD. Stimulated by dental plaque, inflammatory cytokines (such as [interleukin] IL-1β, IL-6, tumor necrosis factor-α) and chemokines (such as MCP-5, IL-8, MIP-1α, prostaglandin E2, and NO) are produced in periodontitis lesions.^20^ These inflammatory mediators can enter the systemic circulation and exert a notable influence on the development of liver diseases, such as NAFLD.^11,25,26^
3. Oxidative stress may contribute to the development of periodontitis and NAFLD. Dos Santos Carvalho et al^12^ investigated whether steatosis and oxidative stress induced by experimental periodontitis could be reversed in the liver. The results showed that the periodontitis group had substantially higher levels of total serum cholesterol, TG, and malondialdehyde (MDA), as well as reduced glutathione (GSH) levels compared to the control and periodontitis recovery groups. They also found that periodontal inflammation could promote the release of inflammatory cytokines, leading to hepatic lipid peroxidation. With the alleviation of periodontitis, the liver damage gradually decreased, which provided evidence for the association between periodontitis and hepatic steatosis.^12^ In addition, Mester et al^27^ established a rat model of periodontitis using ligation. They found that periodontitis could promote hepatic oxidative damage by raising MDA levels and reducing GSH levels. It could also promote hepatic fibrosis by increasing the concentration of matrix metalloproteinase 8 (MMP8) in the serum, gingival tissue, and liver.^27^
4. Dysbiosis of the gut microbiota induced by swallowed oral bacteria (such as *Aggregatibacter actinomycetemcomitans* and *P. gingivalis*) may be a possible pathway connecting periodontitis and NAFLD. *A. actinomycetemcomitans*, which is typically identified in severe periodontitis, is also associated with aggressive periodontitis. Komazaki et al^23^ showed that infection with *A. actinomycetemcomitans* has a negative influence on NAFLD through the alteration of gut microbiota and glucose metabolism. Arimatsu et al^3^ examined the impact of oral administration of *P. gingivalis* on intestinal microbiota and tissue inflammation levels in mice. The researchers observed that mice which had been administered *P. gingivalis* exhibited elevated levels of endotoxins in their bloodstream, a notable decrease in the expression of tight junction proteins in the ileum, and changes in the composition of the intestinal microbiota compared to mice in the healthy control group.^3^ Nakajima et al^29^ discovered that a single oral administration of *P. gingivalis* in mice had a profound influence on the composition of the intestinal microbiota and the permeability of intestinal barrier function. The observed alterations in the intestinal microbiota were closely associated with a decline in the integrity of intestinal barrier function. Consequently, this compromised barrier function allowed a substantial quantity of toxins present in the intestinal tract to enter the liver via the portal circulation, thereby posing a threat to liver health.^29^

Finally, it is necessary to consider the potential confounding effects of smoking and T2DM as common risk factors in the investigation of causality between periodontitis and NAFLD. Previous studies have identified smoking and T2DM as risk factors for both periodontitis and NAFLD.^33,36,39,46^

This study had several strengths. First, the bidirectional MR analysis revealed a more robust association between periodontitis and NAFLD. Second, the MR method provided precise estimates of causal effects by considering confounding factors and reverse causality. Third, sensitivity analyses were conducted to verify the stability and reliability of the obtained causal effect conclusions. However, this study also had some limitations. First, the entirety of the GWAS data was derived only from European ethnicities. Therefore, generalising our findings to other ethnicities would be challenging. MR analyses should be conducted on other populations in the future. Second, the SNPs selected as IVs did not exhibit a strong association with periodontitis (p < 5x10^-6^), which may have affected the statistical power. Third, there may have been sample overlap between the exposure and outcome studies. Fourth, the GWAS data lacked some important variables such as the clinical characteristics of the samples, different types and stages of periodontitis and NAFLD. Therefore, in our investigation, analysing the causal relationship between the two diseases regarding their severity is extremely complex and the results may be biased. Finally, bias from residual and unmeasured confounding factors may have existed despite careful adjustments for multiple confounding factors.

## Conclusion

This study investigated the causality between periodontitis and NAFLD using bidirectional two-sample MR analysis. Our findings showed no genetic evidence for a causal association between periodontitis and NAFLD, suggesting that periodontitis may not have a direct impact on NAFLD progression and vice versa.
